# Malar Bone Metastasis Revealing a Papillary Thyroid Carcinoma

**DOI:** 10.1155/2012/795686

**Published:** 2012-07-17

**Authors:** Ihsen Slim, Aida Mhiri, Imène Meddeb, Aida Goucha, Saïd Gritli, Mohamed Faouzi Ben Slimene

**Affiliations:** ^1^Department of Nuclear Medicine, Salah Azaiez Institut, University El Manar II, 1006 Tunis, Tunisia; ^2^Department of Immunohistocytology, Salah Azaiez Institut, University El Manar II, 1006 Tunis, Tunisia; ^3^Department of Otorinolaryngology, Salah Azaiez Institut, University El Manar II, 1006 Tunis, Tunisia

## Abstract

Papillary thyroid carcinoma is the most common form of differentiated thyroid carcinoma. It is generally confined to the neck with or without spread to regional lymph nodes. Metastatic thyroid carcinomas are uncommon and mainly include lung and bone. Metastases involving oral and maxillofacial region are extremely rare. We described a case of malar metastasis revealing a follicular variant of papillary thyroid carcinoma, presenting with pain and swelling of the left cheek in a 67-years-old female patient with an unspecified histological left lobo-isthmectomy medical history. To our knowledge, this is the first recorded instance of a malar metastasis from a follicular variant of papillary thyroid carcinoma.

## 1. Introduction

Papillary thyroid carcinoma (PTC) is the most common form of thyroid malignancy with a good prognosis, since it is generally confined to the neck with or without spread to regional lymph nodes [1]. However, distant metastases are rare and mainly include lung and bone [2]. Metastasis affecting oral and maxillofacial region is extremely rare, occurring in 0.2‰ of all cases of thyroid carcinoma [3]. We report the first case of a malar metastasis revealing a follicular variant of papillary thyroid carcinoma (FVPTC).

## 2. Case Report

In November 2008, a 67-years-old female was referred to our hospital with the complaint of a slow-growing painless swelling of 6 months duration on her left cheek. She had a medical history of left lobo-isthmectomy of the thyroid gland, seven years ago, with unspecified histology.

Physical examination showed a 5 × 5 cm, firm, and immobile mass of the left malar area without significant lymphadenopathy. The neurological examination and visual acuity were normal.

**Figure 1:**
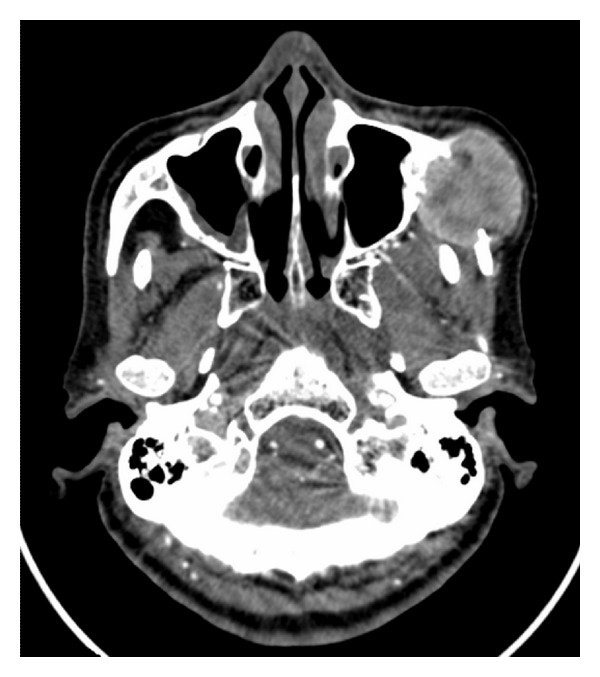
CT of the face with contrast that showed a large enhancing soft tissue mass involving the left malar bone with maxillary wall extension.

A computed tomography (CT) scan showed a 6.0 × 5.5 × 4.5 cm hypervascular soft tissue mass within the left malar region with bony destruction and extension to the lateral walls of the maxilla and the orbit (Figure 1).

An incisional biopsy was performed. The histopathological examination was very suggestive of acinar cell carcinoma of salivary glands. Taking into account her history of thyroid pathology and the absence of a salivary mass, a rereading of the biopsy slides with immunohistochemical studies showed expression of thyroglobulin (Tg) by the tumor cells and confirmed zygomatic bone metastasis of a PTC.

**Figure 2:**
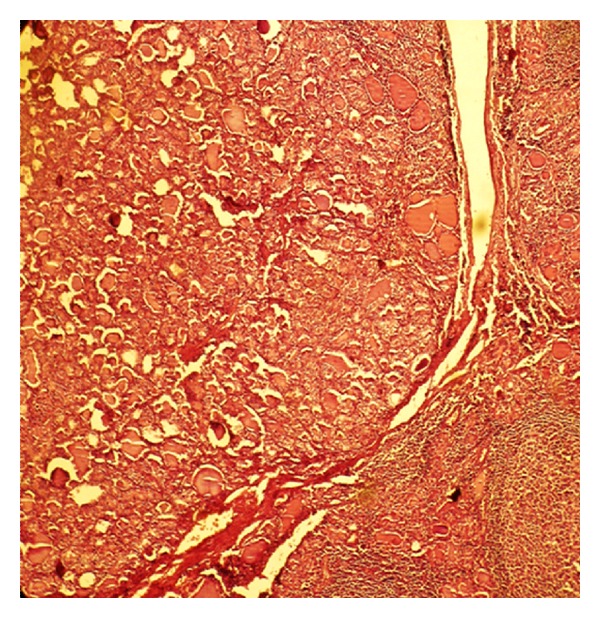
Histologic section of the remaining lobe showing a papillary carcinomatous nodule surrounded by lymphocytic thyroiditis lesions (H&E; original magnification-40x).

**Figure 3:**
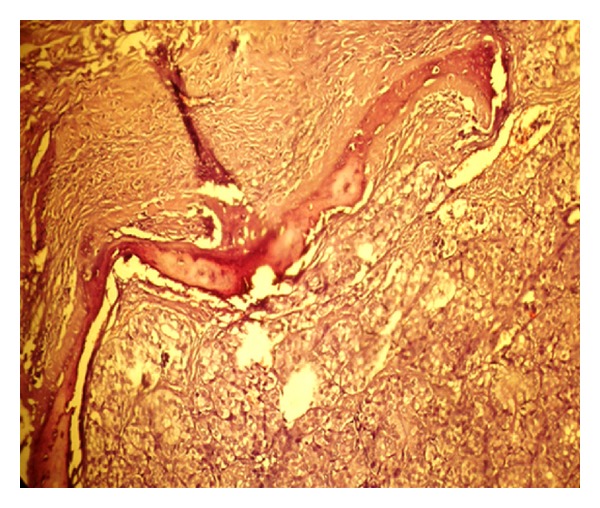
Papillary carcinoma invading the malar bone (H&E; original magnification-20x).

The thyroid ultrasonography revealed an increased right lobe with a 1.6 cm hypoecogenic nodule. Surgical resection was scheduled. Preoperative chest radiographs and subsequent CT showed lung metastases. In February 2009, the patient underwent surgical resection of the malar mass and a simultaneous completion thyroidectomy with central lymph node dissection. Histopathology confirmed the diagnosis of 3 foci of papillary thyroid microcarcinoma in his follicular variant (Figure 2), with metastasis to the malar bone (Figure 3) (TNM classification [6th ed.]: pT1m N1a M1).

**Figure 4:**
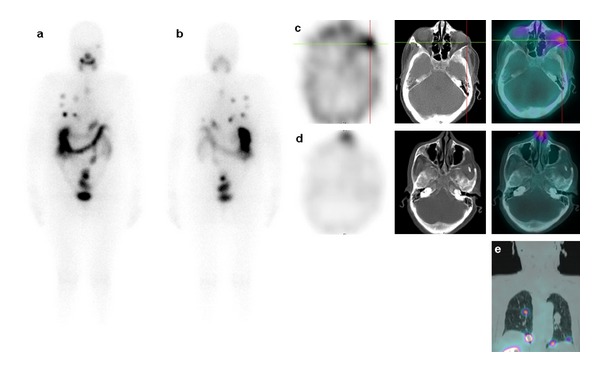
The second post-therapeutic I131 whole-body scan ((a) anterior view; (b) posterior view) showed a regression of the neck foci, an attenuation of the facial uptake, and a stability of lung metastases. SPECT/CT fusion images ((c) and (d): left, SPECT; middle, CT; right, SPECT/CT) established that facial focus corresponded to a remaining circumscribed metastasis of the lateral wall of the left orbit. Coronal SPECT/CT image fusion (d) confirmed micronodular pulmonary metastases.

The patient was then referred to our department for postoperative radioiodine (I131) therapy. She received 5.55 GBq (150 mCi) of I131 after 3 weeks of L-thyroxin withdrawal. Planar total body scintigraphy was performed after 4 days with a mono-head gamma camera (Siemens Integrated Orbiter) showing radioiodine-avid foci in the left cheek region, thyroid bed, and lungs. The TSH-stimulated serum Tg level was 175 ng/mL (absence of anti-Tg antibodies). Six months later, new serum measurement reveals a decreasing of the Tg level (on TSH stimulation) to 18 ng/mL. Patient was given a second high-dose radioiodine therapy (5.55 GBq). Postablative I131 whole-body scan was performed, followed by SPECT/CT scan of the neck and chest, to correlate functional and anatomical images, with a Symbia T camera (Figure 4). Fused images showed a complete attenuation of tracer uptake on the neck and the facial region, except for a focus on the left orbit, with unchanged appearance of the micronodular pulmonary metastases. Being avid to I131, the patient was given two other therapeutic radioiodine cures (2 × 5.55 GBq). The follow-up dignostic I131 scan (March 2012) was negative with undetectable TSH-stimulated thyroglobulin (absence of anti-Tg antibodies). The patient was considered in remission and will be followed by cervical ultrasound, Tg, and diagnostic whole-body I131 scans, on the basis of the department's protocol.

## 3. Discussion

Approximately 90% of malignant thyroid tumors well differentiated and are classified as PTC (80%) or follicular carcinoma (10%) [1]. The Follicular variant of papillary thyroid carcinoma is a major subtype of PTC [4]. Patients with FVPTC and patients with classical PTC showed similar clinical characteristics and prognostic factors. Survival was comparable in both groups [5]. A minority of patients may present, or subsequently develop, locoregional and distant metastases that may adversely affect survival [6]. Distant metastases occur in 4% to 8% of patients with PTC [4–7]. Bone metastasis is the second most common metastatic site after lung. Follicular thyroid carcinoma is known to metastasize via the bloodstream; such a pattern of spread is rare in papillary thyroid carcinoma [8]. The latter exhibits more propensities for lymphatic spread. There have been a few reports of “aggressive” FVPTC that have metastasized hematogenously; these neoplasms have been diffusely invasive or multicentric in the thyroid [9].

Metastatic thyroid carcinoma rarely involves the oral and maxillofacial (OMF) region. They constitute 5.8% of all OMF metastasis, and they are usually located in the jaw bones [3].

According to the literature, the zygomatic bone is a very unusual site for metastasis. We found only 5 cases of metastatic localization from lung, rectum, liver, uterine and breast cancer [10–14]. To our knowledge, this is the first recorded instance of a malar metastasis from FVPTC.

The optimal treatment of such cases may be a simultaneous resection of the solitary bony metastases along with total thyroidectomy followed by radioiodine therapy with subsequent adequate radiotherapy. This may provide a better prognosis because of the enhanced uptake and increased effectiveness of postoperative radioiodine in dealing with other systemic micrometastases that are otherwise undetectable [2, 8].

Common malignancies can metastasize to unusual sites and, although infrequent, may be the presenting feature. Although uncommon, OMF lesions can be the first manifestation of a differentiated thyroid carcinoma. This is the first report of a FVPTC presenting as a malar metastasis. The successful management of such cases may be achieved by a multidisciplinary approach.
